# Health Benefits of Esports: A Systematic Review Comparing the Cardiovascular and Mental Health Impacts of Esports

**DOI:** 10.7759/cureus.40705

**Published:** 2023-06-20

**Authors:** Kofi D Seffah, Korlos Salib, Lana Dardari, Maher Taha, Purva Dahat, Stacy Toriola, Travis Satnarine, Zareen Zohara, Ademiniyi Adelekun, Areeg Ahmed, Sai Dheeraj Gutlapalli, Deepkumar Patel, Safeera Khan

**Affiliations:** 1 Internal Medicine, California Institute of Behavioral Neurosciences & Psychology, Fairfield, USA; 2 Internal Medicine, Piedmont Athens Regional Medical Center, Athens, USA; 3 General Practice, El Demerdash Hospital, Cairo, EGY; 4 Medicine, St. Martinus University Faculty of Medicine, Willemstad, CUW; 5 Pathology, California Institute of Behavioral Neurosciences & Psychology, Fairfield, USA; 6 Pediatrics, California Institute of Behavioral Neurosciences & Psychology, Fairfield, USA; 7 Family Medicine, California Institute of Behavioral Neurosciences & Psychology, Fairfield, USA; 8 Internal Medicine, California Institute of Neuroscience, Thousand Oaks, USA; 9 Internal Medicine, Richmond University Medical Center Affiliated with Mount Sinai Health System and Icahn School of Medicine at Mount Sinai, New York, USA; 10 Internal Medicine Clinical Research, California Institute of Behavioral Neurosciences & Psychology, Fairfield, USA

**Keywords:** sports, anxiety, depression, addiction, gambling, mental health, cardiovascular disease, heart health, esports

## Abstract

Sports all over the world are celebrated and embraced as an indicator of triumph of youth and the human experience. Esports have increasingly come to be associated with an industry likened to traditional sports. Professional gamers who continuously define new standards in the areas of gaming, entertainment, and esports have emerged. This systematic review sought to find out the extent to which these virtual sports affect cardiovascular and mental health, both positively and negatively, and if this is comparable to traditional sports to any degree. Using the Preferred Reporting Items for Systematic Reviews and Meta-analyses, we reviewed journals and full-text articles that addressed the topic with keywords, such as esports, cardiovascular, mental health, gaming, and virtual reality. Six articles were selected after quality assessment. In summary, rehabilitative medicine currently benefits the most from this entertainment platform, with comparable findings in the positive and negative effects on mental health. Cardiovascular health appears to benefit from esports, with an increase in physical activity with use, but is not at the level of replacing traditional sports. Unlike as seen with traditional sports, addiction to gaming appears to be a steadily emerging issue that mental health practitioners will, in the not-so-distant future, have to lay ground rules for if esports are to be incorporated in everyday affairs.

## Introduction and background

Electronic sports (esports) are gamified interactions propelled by electronic modules in which participants interact through a computer intermediary [1]. These interactions may be collaborative or competitive. When individuals or groups compete against one another, with a defined set of rules, we deem this a game. Games are played, developed, and won with tactics and strategies, which increase cognitive flexibility [2]. Collaborative sports, such as rowing, hockey, and soccer, on the other hand, provide another unique set of social skills. Typically, concepts, such as fan bases, material rewards, fitness, and training styles, have been associated with traditional sports of all kinds. Today, like many collaborative and competitive endeavors around the world, esports is growing and gaining attention with viewership [3]. It has, over the years, come to be incorporated into sports festivals around the globe. Between 2018 and 2021, there were over 400 million viewers of esports worldwide, with viewership expected to continuously rise in the coming years. The pandemic is notably an enabler in the rise of this trend. It is predicted that the total earnings of players around the world from esports will exceed US$500 million by the end of 2023 [4].

Esports are reported to improve reflexes and eye-hand coordination, although data appear mixed [5]. Memory, attention, and awareness are noted to be enhanced by some of these games [6]. At the onset of the COVID era, esports provided a sense of community and engagement to players and participants, although this growth was accompanied by increased threat to cybersecurity and intellectual property [7]. As opposed to traditional sports, esports seem to come with unique drawbacks, with regard to health. Long-term users of video games report eye strain and a higher frequency of refractive errors [8]. Some games are associated with high stress levels and burnout. Video games may also disrupt sleep patterns [9].

This article takes note of the above information as it pertains to esports and video games. We note that there is a gap in knowledge with regard to esports and cardiovascular health. While other health modalities can boast of improving cardiovascular indices, it is hard to rule esports in or out in this regard. Do esports cause detriments to the cardiac function in the long run? Are there any benefits or advantages to cardiovascular morbidity or mortality when it comes to esports? Are effects purely user dependent? Moreover, we seek to find out the effects of esports on mental health. Much has been reported that is related to addiction, betting, depression, and anxiety. This paper seeks to find out if these devices are indeed tools that may be channeled to improve mental well-being or if they are a source of impending problems.

## Review

Methods

Reporting Guideline

This systematic review was written according to the Preferred Reporting Items for Systematic Reviews and Meta-Analyses (PRISMA) 2020 guidelines [10].

*Database and Search Strategy*

Our search was initiated between February 12, 2023 and February 19, 2023. The following databases were used as a part of our search: PubMed, MedLine, PubMed Medical Subject Heading (MeSH), ResearchGate, ScienceDirect, and Science.gov. Keywords chosen for the search include "eSports," "health," "mental health," "cardiovascular health," "computer games," "video games," "virtual reality," and "exergames." In addition to the above keywords, the following was employed in the search using PubMed MeSH ((((("Health"[Mesh]) AND "Virtual Reality" [Mesh]) AND "Sports"[Mesh]) OR ( "Virtual Reality Exposure Therapy"[Mesh] OR "Exergaming"[Mesh] )) OR ( "Exergaming/injuries"[Mesh] OR "Exergaming/physiology"[Mesh] OR "Exergaming/psychology"[Mesh] )) AND ( "Video Games/adverse effects"[Mesh] OR "Video Games/psychology"[Mesh] ). Searches were conducted with the help of Booleans operators AND, OR, in various search engines of the databases, limiting findings to papers written between 2017 and 2023. Table 1 is a summary of our search strategies.

**Table 1:** Databases and search strategies used MeSH: Medical Subject Heading

Database	Search strategy	Number of papers
PubMed/Medline	Esports AND health	35
PubMed MeSH	("Health"[Mesh]) AND "Virtual Reality"[Mesh]) AND "Sports"[Mesh]) OR ( "Virtual Reality Exposure Therapy"[Mesh] OR "Exergaming"[Mesh] )) OR ( "Exergaming/injuries"[Mesh] OR "Exergaming/physiology"[Mesh] OR "Exergaming/psychology"[Mesh] )) AND ( "Video Games/adverse effects"[Mesh] OR "Video Games/psychology"[Mesh] )	10
ResearchGate	Esports AND health AND cardiovascular AND mental	18
ScienceDirect	Esports AND health benefits AND computer games AND virtual gaming AND mental health AND cardiovascular health AND computer games	52
Science.gov	Esports AND health AND virtual games AND competition AND cardiovascular health AND mental health	13

Inclusion and Exclusion Criteria

We were interested in all individuals, regardless of age, who used a human-computer interface or an electronic game, to achieve both sport and non-sport endpoints. We believe that this also satisfactorily embraces the domain of esports, in being goal-oriented, either collaborative or competitive, with clearly set expectations. All individuals who patronize esports as users of video games or spectators, for any length of time or duration, for any reason, be it professional or recreational, were included in the population study. Studies selected were full texts, regardless of the study style or type. Assessing the extent to which esports can replace traditional sports by way of health benefits was the main outcome of interest. The health outcomes of esports against traditional sports, which generally involves whole-person involvement and more physicality, were investigated. More succinctly, the role of esports on the cardiovascular, mental, and overall health was compared to that of traditional sports. We were not interested in the degree of usage/patronage as much as the experience following usage and the impact on cardiac and mental health. Table 2 further summarizes our criteria.

**Table 2:** Inclusion and exclusion criteria

Inclusion criteria	Exclusion criteria
All individuals who have participated in esports	Grey literature
Full-text articles that have addressed esports and health, in particular mental and cardiovascular health	Unpublished data
Published within the last six years (2017-2023)	Literature not in the English language
Published in the English language or satisfactorily translated from another language into English	Opinion texts
No age restrictions	

Screening of Articles

**Figure 1:**
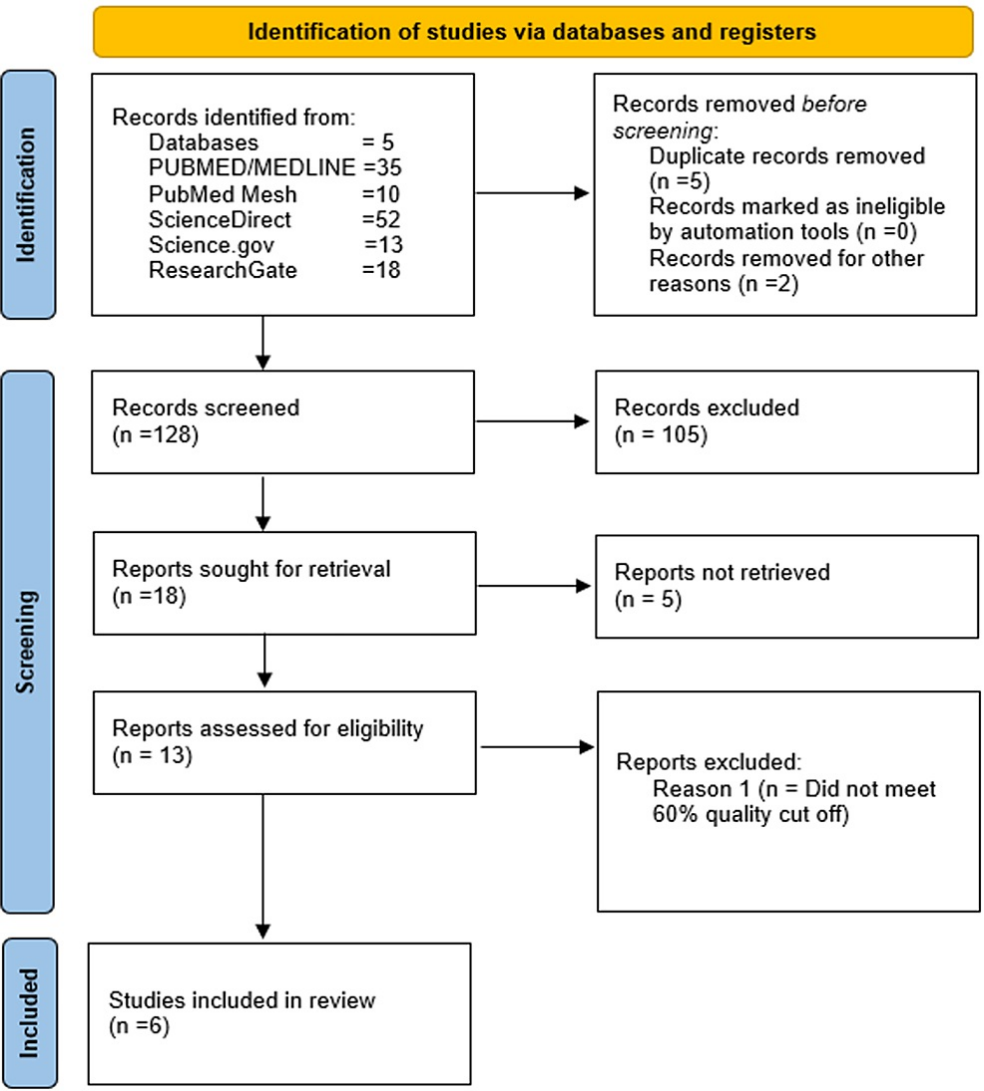
PRISMA checklist for the final selection of publications PRISMA: Preferred Reporting Items for Systematic Reviews and Meta-Analyses

After obtaining articles based on the above criteria, two lead authors further narrowed them down based on the scope of the study. Conclusions, abstracts, titles, and method sections were reviewed at this phase. Duplicates were manually identified and deleted. The resultant selection was then subjected to quality assessment. Figure 1 demonstrates our screening process.

Quality Appraisal

Two independent investigators (the first and second authors) performed article selection, assessment, and analyses in each step. If there was a contradictory result regarding an article’s eligibility, its full text was reassessed by consensus within the group. A Measurement Tool to Assess Systematic Reviews (AMSTAR) checklist was used in assessing both systematic reviews and meta-analyses. The Newcastle-Ottawa classification tool was used in assessing cross-sectional studies. The Joanna Briggs Institute (JBI) checklist was used in assessing case reports. Only studies with a quality appraisal of 60% and above were selected for the final evaluation. Table 3 highlights the quality appraisal tools employed in this study.

**Table 3:** Quality appraisal tools employed in the study AMSTAR: A Measurement Tool to Assess Systematic Reviews; JBI: Joanna Briggs Institute

Study type	Quality appraisal tool
Systematic reviews/meta-analyses	AMSTAR checklist
Cross-sectional studies	Newcastle-Ottawa classification tool
Case reports	JBI checklist

Results

As this review was designed to be a mixed-methods study, we found it more pragmatic to work with a purely systematic review, without an additional meta-analysis. Studies in the area are largely observational, with little recourse to blinding, as this was noted by various research groups to be a limiting factor, nearly impossible or even unethical, in administering devices to individuals without at least a partial disclosure. This affected the study designs and outcomes, but this was not unexpected. A total of eight systematic reviews and meta-analyses were sampled in the selection phase. Of these, three were selected following quality assessment [11-13]. Table 4 summarizes the quality appraisal process for the systematic reviews.

**Table 4:** AMSTAR checklist of articles that met the criteria for the systematic review AMSTAR: A Measurement Tool to Assess Systematic Reviews; PICO: population, intervention, control, and outcomes

AMSTAR criteria	Chen et al. [11]	Sardi et al. [12]	Yu and Chan [13]
Did the research questions and inclusion criteria for the review include the components of PICO?	Yes	No	No
Was a “priori” design implemented?	Yes	Yes	No
Did the review authors explain their selection of the study designs for inclusion in the review?	Yes	Yes	Yes
Did the review authors use a comprehensive literature search strategy?	Yes	Yes	Yes
Did the review authors perform study selection in duplicate?	Yes	Yes	Uncertain
Did the review authors perform data extraction in duplicate?	Yes	Uncertain	Uncertain
Did the review authors provide a list of excluded studies and justify the exclusions?	No	No	No
Did the review authors describe the studies included in adequate detail?	Yes	Yes	No
Did the review authors use a satisfactory technique for assessing the risk of bias in individual studies that were included in the review?	Yes	Yes	Yes
Did the review authors report on the sources of funding for the studies included in the review?	Yes	Yes	Yes
If a meta-analysis was performed, did the authors use appropriate methods to statistically combine results?	Yes	No	Yes
If a meta-analysis was performed, did the review authors assess the potential impact of risk of bias in individual studies on the results of the meta-analysis or other evidence synthesis?	Yes	No	Yes
Did the review authors account for risk of bias in individual studies when interpreting/discussing the results of the review?	Yes	Yes	Yes
Did the review authors provide a satisfactory explanation for and discussion of any heterogeneity observed in the results of the review?	Yes	Yes	Yes
If they performed quantitative synthesis, did the review authors carry out an adequate investigation of publication bias (small study bias) and discuss its impact on the results of the review?	No	No	Yes
Did the review authors report any potential sources of conflict of interest, including any funding they received for conducting the review?	Yes	Yes	Yes
Total score (out of 16)	14/16	10/16	10/16
Overall methodological quality	Accepted 87.5%	Accepted 62.5%	Accepted 62.5%

A total of five cross-sectional studies were brought under review. Following the quality assessment, two articles remained for the review. Table 5 summarizes the appraisal process for the cross-sectional studies.

**Table 5:** Quality appraisal using the Newcastle-Ottawa classification tool for cross-sectional studies *Demonstration of degree of approval per guidelines in the Newcastle-Ottawa classification tool

Article	Representativeness *	Sample size *	Non-respondents *	Ascertainment of exposure **	The subjects in different outcome groups are comparable, based on the study design or analysis. Confounding factors are controlled. **	Assessment of the outcome *****	Statistical test *	Long follow-up	Adequacy of follow-up	Accept/Reject (%)
Lelonek-Kuleta and Bartczuk [14]	*	*	*	*	*	****	*	-	-	Accept 67%
Soares et al. [15]	-	*	*	*	**	***	*	-	-	Accept 60%

One report was reviewed using the JBI checklist and was accepted [16]. Table 6 summarizes our findings per the checklist.

**Table 6:** Quality assessment of the case report Source: Niedermoser et al. [16]

Question	Answer
Were the patient’s demographic characteristics clearly described?	Yes
Was the patient’s history clearly described and presented as a timeline?	Yes
Was the current clinical condition of the patient on presentation clearly described?	Yes
Were the diagnostic tests or assessment methods and the results clearly described?	Yes
Was the intervention(s) or treatment procedure(s) clearly described?	Yes
Was the post-intervention clinical condition clearly described?	Yes
Were adverse events (harms) or unanticipated events identified and described?	Yes
Does the case report provide takeaway lessons?	Yes

Table 7 is a summary of articles selected for the final review in this publication. Categories were itemized as areas of interest in this paper, that is, cardiovascular and mental health. Findings were also summarized to reflect the areas of interest, often reflecting conclusions drawn by respective authors.

**Table 7:** Summary of all articles by author, year of publication, and emphasis within the study

Authors	Year of publication	Paper type	Number of participants/number of articles reviewed	Focus of paper (cardiovascular/mental health)	Summary of findings
Chen et al. [11]	2022	Systematic review and meta-analysis	10 articles	Cardiovascular health	Virtual reality improves physical activity and reduces depression.
Sardi et al. [12]	2017	Systematic review.	46 articles	Cardiovascular health, mental health	E-games may be used in health in the areas of chronic disease rehabilitation, physical activity improvement, and mental health.
Yu and Chan [13]	2021	Meta-analysis	31 articles	Cardiovascular health, mental health	Some devices (mouse/keyboard) are superior to others in the development of cognition.
Lelonek-Kuleta and Bartczuk [14]	2021	Cross-sectional study	2074 participants	Mental health	Modifiable behavior tools have a large role to play in gambling addiction.
Soares et al. [15]	2022	Cross-sectional study	401 participants	Mental health	Rules for engaging in esports with regards to COVID-19 need to be formalized.
Niedermoser et al. [16]	2021	Case report	1 individual	Mental health	Cognitive behavioral therapy is effective in treating non-substance addiction.

Discussion

Esports and Cardiovascular Disease: Benefits

Increasingly, all over the world, esports is gaining popularity and momentum. Going further, some international sports events are introducing virtual tournaments as a part of their content [17]. A form of these sports, called exergames, aims to increase physical activity and promote cardiometabolic health [17]. Moreover, the use of wearable electronic devices has been associated with increased motivation for health-seeking behavior and cardiovascular health as a whole [18]. The awareness provided through electronic media remains vital, as amateur and professional gamers will have to incorporate traditional methods into their daily activities in order to obtain optimal outcomes [19]. In addition, the platforms that host these games are increasingly being used to promote awareness of healthy habits, including ergonomic tips and campaigns against smoking. Examples of gaming devices associated with improved health include motion sensing controllers, such as mats, boards, and gloves [20]. In addition, casual video games and even exergames have been identified as stress-reducing activities, overall, when used in the right manner [21]. Stress reduction is key in preventing adverse cardiovascular outcomes. Our studies show that virtual reality games, an increasingly popular platform for gaming, have the potential to promote physical activity based on the design of the game. Exercise capacity was noted to improve with virtual reality-guided training methods in one study. This was particularly true for those undergoing cardiac rehabilitation. The study went as far as noting improvements in total cholesterol and low-density lipoprotein as a benefit of engaging in virtual sports [11]. The idea of rehabilitation was further buttressed by Sardi et al. [12], when addressing the role that virtual sports may play in mainstream health. Conclusively, both studies that tackled the domain on cardiovascular health and esports/virtual gaming noted improvement in physical activity as a clear benefit [11,12].

Esports and Cardiovascular Diseases: Harms

We maintain that the effects of gaming are both in-game and after-game. In-game systolic blood pressure and heart rates were considerably higher in gamers during play and could potentially be a trigger for adverse outcomes in patients with borderline or established heart diseases [22]. In contrast to traditional sports, esports is not associated with the expenditure of energy nor the metabolic benefits derived from the former [23]. Although, anecdotally, games like chess have been linked with the burning of calories, quoted at up to about 6000 calories a day [24], it is hard to tell whether the physical demands and long hours of training and decreased calorie intake in preparation for games contribute to these calorie losses or if these losses are purely from the intensity of a single bout. By contrast, sedentary lifestyles and physical inactivity, both associated with chess in particular and esports as a whole, are a leading risk factor for cardiovascular diseases and all-cause mortality [25,26]. Our sampled studies highlighted the role of esports, consoles, and video games in promoting physical activity especially in the area of rehabilitation, including physical and cardiovascular [11-13]. It appears that the intent, design, and execution of these devices and software decide for the most part how far the cardiac gains or harms play out. Highlighting both harms and benefits to the heart, the verdict remains uncertain, as to whether or not to consider esports and virtual games as actual sports [27]. At this time, it would be premature to consider esports a suitable and complete substitute for traditional sports and exercise. Indeed, it is recommended that professional and amateur gamers incorporate regular physical activity in non-gaming mode/traditional activity in order to enhance their gameplay and promote their overall health [25].

Esports and Mental Health: Benefits

The virtual platform has been associated with stress relief, skill-building, improved resilience, improved attention and focus, and a reduction in risks of depression, anxiety, and related mental health disorders [11-13]. The role of esports in social connectedness and resilience was highlighted by the COVID-19 pandemic, when more engagement was fostered using these platforms worldwide [15]. However, many of these benefits may be viewed as side benefits while enjoying the game. Its role in rehabilitation, on the other hand, appears to require intentional design [12]. Moreover, the benefits appear more noticeable in controlled or supervised settings [28]. We will not discount the role of the Hawthorne effect in this finding. In addition, device use limitation may be associated with better outcomes in terms of the positives outlined above [16,28]. Overall, targeted use of esports for a specific domain of mental health may hold benefits comparable to those of traditional sports [29]. Other benefits worth highlighting include improved eye-hand coordination, spatial awareness, attention, and focus, depending on the game and in-game competencies [30]. As things stand, formal training methods are at the inchoate stage for professional gamers [30].

Esports and Mental Health: Harms

Addiction remains of great concern in the engagement of online sports. Even regular, non-professional gamers are at risk. Non-substance addiction has gained attention in medical circles and is of growing concern [16]. The elements driving these behaviors may largely be driven by tools incorporated into the software [14], although addiction in general has heritable features [31]. There appears to be a relationship between screen time and negative outcomes, such as eye strain, poor posture, sedentary lifestyle, anxiety, and depression [29]. It goes further. There is a negative correlation between device use and sleep duration, with more screen time yielding poorer sleep quality [32]. In addition, professional players are exposed to similar amounts of psychological stress as witnessed among traditional sportsmen [29]. It is interesting to note that the problems created by this new avenue of entertainment are solved by more established, old therapies [16].

Esports and Other Areas of Health

There is ample evidence to support the notion that esports benefit musculoskeletal health, rehabilitation, and recovery. They may serve as the stopgap in recovery and help stall frailty [11-13]. Cognitive benefits have also been underscored [13]. A professional esports player must however pay attention to musculoskeletal health, eye health, nutrition, and sleep and has to purposefully maintain social connectedness, lest run the risk of long-term decline with chronic illness [16,33]. Financial health is of great concern to those with addiction, who may have no guidance as to the demands of their ambition and what it takes to achieve the professional status [3,16].

The Future of Esports and Health

For a rapidly emerging international platform, esports may benefit from better regulations, for the sake of player health, cybersecurity, and the protection against marketers [4]. As more is understood about our behaviors around these sources of entertainment, health warnings and limits may be developed to curb non-substance addiction [14,16]. Beyond therapy and rehabilitation, more entertainment-directed games may be developed, engineered free of craving and addictiveness, as we master and understand the drivers of gambling and addiction that surround these devices [14].

Limitations

This study is a systematic review of the various components of cardiovascular and mental health importance related to esports. Nonetheless, it has limitations: First, no measurable extents of such findings are provided in this article. For this reason, we are unable to say emphatically to what extent the various findings play a role, what the interplay of these findings yields, and whether there are confounders to these findings. While some findings, such as improvement in physical activity and increased risk of non-substance addiction, have been serially replicated and hence may be deemed credible, other findings, such as effects on depression, share contrasting views that this article is ill equipped to address. Second, our paper did not address other domains of healthcare, besides mental and cardiovascular health. Third, we will not downplay the role of the Hawthorne effect in the few positives recorded in both the cardiovascular and mental health domains. Areas for further studies include the therapeutic role of virtual gaming on pulmonary and neurological health and development.

## Conclusions

While producers of esports and gaming tools have the goal of marketing and selling their content, consumers remain exposed to varying degrees of cognitive stimulation, whose effects on other specific domains of health continue to be uncovered. We sought to highlight the role of esports in cardiovascular and mental health. We wanted to find out, objectively, if heart health stood to benefit long term from these devices and their content. We also sought to find out if mental health stood to gain from continued use of gaming devices. Our findings show that there are specific domains of rehabilitation and physical therapy that benefit from such device use. Targeted at recovery, esports serve as excellent tools of engagement in rehabilitative medicine. There appears to be more potential for therapeutics, granted that the right legislature, regulations, and funding are directed at this. Sadly, esports however are not a suitable substitute for traditional sports in the domain of cardiovascular health. Mental health shows comparable benefits from esports as with traditional sports. Professional gamers are exposed to the considerable risk of increased all-cause mortality by engaging in esports. Unfortunately, what constitutes "adequate safety measures" has not been universally agreed upon. Addiction prevention, eye health, hearing health, mental health, and cardiovascular health domains host only arbitrary rules and guidelines from the esports community. In conclusion, these devices are tools, and tools will remain as useful as we decide to make them. Moreover, although these devices and platforms have come to stay, the onus is on proponents and consumers to arm themselves and their dependents from the harms.
